# Purinergic Receptors on Oligodendrocyte Progenitors: Promising Targets for Myelin Repair in Multiple Sclerosis?

**DOI:** 10.3389/fphar.2020.629618

**Published:** 2021-01-27

**Authors:** Davide Lecca, Maria P. Abbracchio, Marta Fumagalli

**Affiliations:** ^1^ Department of Pharmaceutical Sciences, Università degli Studi di Milano, Milan, Italy; ^2^ Department of Pharmacological and Biomolecular Sciences, Università degli Studi di Milano, Milan, Italy

**Keywords:** purinoceptors, GPR17, oligodendrocyte progenitors, myelin repair, multiple sclerosis

## Introduction

Multiple sclerosis (MS) is an inflammatory immune-mediated disease of the central nervous system (CNS) characterized by damage of myelin-forming oligodendrocytes and destruction of myelin itself, leaving denuded axons without trophic and metabolic support and prone to degeneration (Nave, 2010). Clinically, this results in neurological disability and progression from the relapsing-remitting form of the disease to the irreversible chronic progressive one (Franklin and ffrench-Constant, 2008). Current therapies are immunomodulatory drugs that efficiently reduce the number and severity of debilitating immune-mediated attacks at initial stages (Comi et al., 2017), but are not effective in the presence of extensive axonal degeneration. Therefore, early therapeutic strategies promoting the formation of new oligodendrocytes and myelin sheaths around demyelinated axons are highly needed (Franklin and ffrench-Constant, 2017). Importantly, the major source of new myelinating oligodendrocytes, i.e., NG2-glia, traditionally defined as oligodendrocyte precursor cells (OPCs) (Nishiyama et al., 2009), do persist and slowly proliferate in the adult CNS (Dawson et al., 2003). Spontaneous remyelination occurs in MS patients but eventually fails due to several reasons (Franklin and ffrench-Constant, 2008). First, for remyelination to occur, it is important to have functionally healthy axons (Stangel et al., 2017) likely releasing myelination signals. Second, a remyelination supportive environment is required, i.e., a correct balance between different cell types (astrocytes, microglia, macrophages, and other immune cells) (Lombardi et al., 2019; Molina-Gonzalez and Miron, 2019) and mechanisms, including debris phagocytosis and secretion of growth signals or inhibitory molecules, from transcription factors to extracellular (ECM) proteins (Marangon et al., 2020). Finally, successful differentiation of OPCs also depends on their intrinsic potential which is, in turn, strictly dependent on regional heterogeneity (Marques et al., 2016).

Continuous communication between OPCs, neurons, and other glial cells is crucial to regulate both developmental myelination and myelin dynamics during adulthood. In this respect, ATP emerges as an important signaling molecule profoundly influencing OPCs, and functional purinoceptors are found to be expressed on these cells (Fields and Stevens, 2000). Extracellular ATP can be degraded to ADP or adenosine by ectonucleotidases expressed on the cell surface and differently activate purinergic membrane P1 and P2 receptors: the former are G protein-coupled receptors selectively activated by adenosine, whereas the latter are further divided into P2X ionotropic receptors, exclusively activated by ATP and G protein-coupled P2Y receptors with very specific pharmacological profiles (Alexander et al., 2019). For example, P2Y_1_ is primarily activated by ADP and only partially by ATP. This complexity highlights how a single molecule can produce a wide variety of downstream signaling in terms of proliferation, differentiation, migration, response to damage, and cell death, based on the actors involved at cellular and molecular levels. Of note, the expression of some purinoceptors is strictly dependent on specific differentiation stages, indicating critical roles in OPC maturation and myelination. Some of these receptors are also altered under demyelinating conditions, which can itself contribute to disease development (Fumagalli et al., 2016).

In this opinion, we discuss key questions that still remain unclear regarding the involvement of purinergic signaling in NG-glia response. First, what is the function of purinoceptors in OPCs, and do they exert peculiar roles in their behavioral heterogeneity? Second, how do purinoceptors control OPC reactive response to myelin injury? Are their expression and roles altered during the course of MS? Third, would purinergic strategies to promote remyelination by NG-glia be of therapeutic interest?

## Expression and Role of Purinoceptors in Oligodendrocyte Progenitors

Confocal calcium imaging on OPCs from optic nerve demonstrated that ATP evokes rapid and transient increases in [Ca^2+^]_i_, mainly through P2Y_1_ (Hamilton et al., 2010). P2Y_1_ activation also promoted OPC chemotaxis in both isolated OPCs and cerebellar slices (Agresti et al., 2005). ATP-evoked Ca^2+^ signals in both OPCs and mature oligodendrocytes are also mediated by P2X7. However, P2Y_1_ may play a greater role under physiological conditions, being activated at nanomolar ATP concentrations, whereas only upon cell rupture do ATP extracellular levels become millimolar, i.e., high enough to activate P2X7 (Hamilton et al., 2010). In a similar way to NMDA receptors, after prolonged activation, P2X7 promotes the formation of a large nonselective pore enabling leakage of ions, metabolites, and ATP itself, ultimately causing cell death (Matute et al., 2007) and fostering a vicious cycle that activates P2X7 in nearby cells.

The very efficient degradation of extracellular ATP by ectonucleotidases prevents the activation of P2X7-mediated danger pathways and enables a further level of signaling through P1 receptors. Adenosine was initially described to inhibit OPC proliferation and promote OPC differentiation and myelin formation (Stevens et al., 2002). However, the presence of all four P1 receptors, whose expression is timely regulated during differentiation, makes adenosine a more complex modulator.

Adenosine regulates the transition from proliferating OPCs to mature cells in a cAMP-regulated manner. Outward K^+^ currents, essential in early OPCs, are abolished by the selective A_2A_ receptor agonist CGS21680 that blocks their differentiation by stimulating adenylyl-cyclase activity (Coppi et al., 2013). Similarly, the A_2B_ selective agonist BAY60-6583 stimulates cAMP production and inhibits K^+^ currents, thus depolarizing OPC membranes and blocking cell maturation. A_2B_ stimulation also elevates levels of sphingosine-1-phosphate (S1P), a bioactive lipid mediator, contributing to delayed maturation (Coppi et al., 2020). The cross-talk between adenosine signaling and S1P may be clinically relevant because receptors for S1P are targeted by fingolimod, a widely used drug for MS, and whose direct action on the CNS remains unclear (Soliven et al., 2011). In contrast, both the prodifferentiating and promyelinating effect of adenosine on OPCs are likely due to activation of A_1_ receptors inhibiting cAMP (Coppi et al., 2015). The physiological role of A_3_ receptors has not been described, but in optic nerve-derived OPCs, its stimulation with the specific agonist 2-CI-IB-MECA induced apoptosis (González-Fernández et al., 2014).

Although uracil-nucleotides are produced and released in brain tissues, and their receptors are present on both neurons and glia, the contribution of P2Y_2_, P2Y_4_, P2Y_6_, and P2Y_14_ in OPCs has been poorly investigated. In 2006, we described the G protein-coupled receptor GPR17 as a P2Y-like receptor, based on its pharmacological response to UDP, UDP-glucose, and UDP-galactose (Ciana et al., 2006). In physiological conditions, GPR17 is almost exclusively expressed in oligodendrocytes, with a clearly characterized transient pattern: it starts to be expressed in early OPCs, reaches its peak in immature oligodendrocytes, and then disappears in myelinating oligodendrocytes (Fumagalli et al., 2011). Of note, transcriptome analyses revealed that GPR17 clearly characterizes a population of differentiation-committed precursors (Marques et al., 2016) and predominantly labels OPCs within axodentritic area, potentially able to myelinate axons (Marisca et al., 2020).

In OPCs, UDP-glucose promoted maturation to MBP-positive cells, whereas cangrelor, a nonselective GPR17 antagonist, maintained cells at an undifferentiated stage (Fumagalli et al., 2011). The prodifferentiative effect of GPR17 agonists may be due to receptor desensitization and internalization: prolonged activation of GPR17 promotes its removal from the membrane, thus enabling terminal maturation (Fratangeli et al., 2013). In purified primary OPCs, UDP-glucose stimulated cell migration and enhanced outward K^+^ currents (Coppi et al., 2013). In both transfected cell lines and primary OPCs, uracil-ligand-evoked responses were antagonized by Cangrelor and MRS2179, two purinergic antagonists (for review, see Lecca et al., 2020).

## Dysregulation of Purinoceptors under Disease Condition and Their Potential as Therapeutic Targets

In human MS and animal models, P2X7, GPR17, and adenosine receptors undergo significant changes. Specifically, postmortem analysis of human MS specimens revealed P2X7 increases in optic nerve oligodendrocytes and in activated microglia and astrocytes in both spinal cord and brain (Amadio et al., 2017). In experimental autoimmune encephalomyelitis (EAE) mice, a model reproducing several features of human MS, upregulated P2X7 was described in both activated microglia and astrocytes already during the asymptomatic phase and in oligodendrocytes and neurons after disease onset (Matute et al., 2007; Grygorowicz et al., 2010). The adenosine produced after ATP breakdown may exhibit anti-inflammatory and immunosuppressive actions by inhibiting T-cell proliferation and cytokines secretion (Saze et al., 2013), but ATP signaling is overwhelming and generates an amplification cascade that eventually kills oligodendrocytes. After disease onset, administration of oxATP reduced clinical outcomes and demyelination extent (Matute et al., 2007), likely acting on astroglial and microglial P2X7, supporting the hypothesis that P2X7 receptor blockers could have a role in preventing/improving MS symptoms. Due to its peculiar pharmacology, P2X7 acts as a “silent” receptor whose activation takes place only under pathological conditions (Bhattacharya and Biber, 2016). Thus, P2X7 inhibitors are not expected to significantly affect physiological receptor activity and may become ideal candidate drugs in treating inflammation in a pleiotropic manner. However, despite several specific P2X7 antagonists have been developed so far, improving pharmacokinetics and blood–brain barrier (BBB) permeability, only a few of them entered clinical trials for peripheral diseases, such as rheumatoid arthritis and chronic obstructive pulmonary disease. The safety on humans was confirmed, but the efficacy was disappointing in most cases, potentially due to different factors, including the limits of the animal models, the presence of several P2X7 haplotypes in humans, and the lack of knowledge about their actual contribution in disease pathogenesis. Moreover, some of the candidate molecules were noncompetitive allosteric modulators that may not be sufficient to inhibit massive P2X7 activation. (for review, see Di Virgilio et al., 2017; Calzaferri et al., 2020).

GPR17 is a relatively novel receptor. Its almost exclusive expression in OPCs gives a new opportunity to target these cells and enhance their remyelination capabilities in damage conditions. In chronic damage and inflammation, GPR17 becomes pathologically overexpressed, which prevents cells’ terminal maturation. Lack of GPR17 timely downregulation was described in several animal models, resulting in impaired myelination (Lecca et al., 2008; Chen et al., 2009; Coppolino et al., 2018). Antagonists were indeed effective in preventing acute damage in a model of brain ischemia (Ciana et al., 2006; Lecca et al., 2008), but chronic administration of receptor antagonists in MS should be carefully evaluated to avoid unexpected side effects in oligodendrocyte functions in terms of metabolic support to neurons or interaction with other cells (Lecca et al., 2020). Conversely, receptor internalization mediated by agonists may promote GPR17 removal from the membrane, thus enabling OPCs to resume maturation. In this respect, promising results have come from *in vivo* study where the selective GPR17 agonist galinex was proved to significantly retard EAE induction (Parravicini et al., 2020). Since inflammation contributes to sustaining GPR17 expression (Coppolino et al., 2018), cotreatment with anti-inflammatory agents may efficiently favor remyelination.

As mentioned, adenosine receptors play crucial roles in OPC differentiation, and their pharmacological modulation may foster remyelination. Despite completely different etiology compared to MS, Niemann–Pick type C 1 (NPC1), a genetic disease characterized by lysosomal accumulation of cholesterol and sphyngolipids, is also characterized by neurodegeneration, neuroinflammation, and dysmyelination. As in MS, in NPC1, OPCs are blocked in immature stages and do not undergo maturation. In a mouse model of NPC1, administration of CGS21680, a selective A_2A_ agonist, rescued OPC maturation (De Nuccio et al., 2019), apparently in contrast to the *in vitro* results described above. Further studies should be encouraged to interpret these conflicting data.

## Conclusive Discussion

**FIGURE 1:**
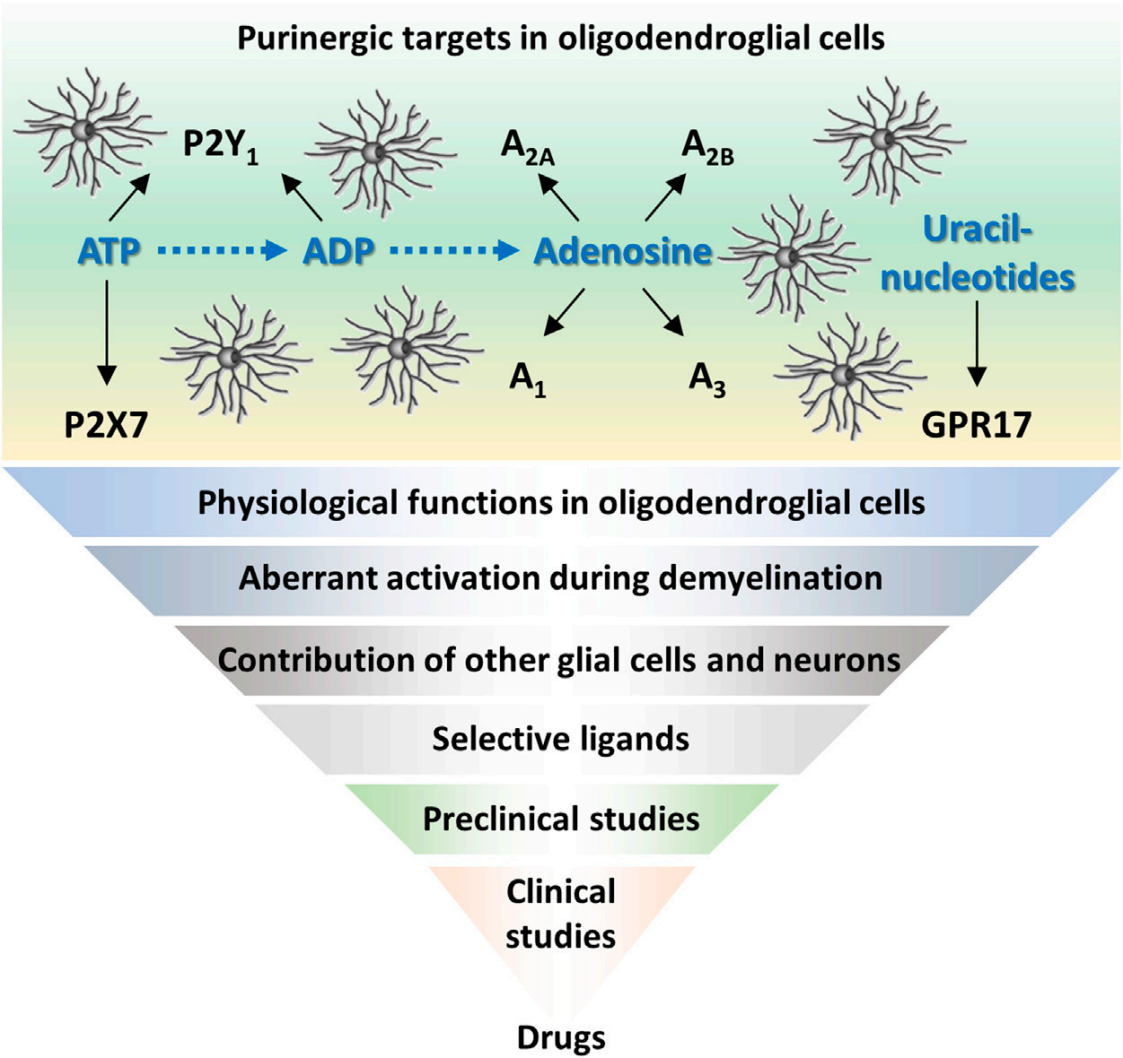
Purinergic receptors in oligodendrocyte progenitors as promising targets in multiple sclerosis. Schematic representation of purinergic receptors expressed in oligodendroglial cells and their ligands. To develop new purinergic-based drugs for remyelination, further efforts should be done in basic research, to provide robust data to be translated into clinics.

Data discussed above suggest that P2X7, GPR17, and adenosine receptors could be valuable targets to stimulate myelin repair, preventing chronic axonal degeneration. For all these receptors, promising molecules have been tested in preclinical studies, but further studies are needed to prove their efficacy in a context close to human paradigms, with minimal or absent interferences with physiologically essential pathways (Figure 1). To reach cells inside the CNS, these compounds should also be able to cross BBB. Although MRI-based gadolinium studies showed leaky BBB during acute MS relapses, evidence indicates that BBB is then re-established. Thus, besides developing brain permeable molecules (as already done for P2X7 antagonist), other approaches should be considered. While virus-based CNS drug delivery to maximize tropism for oligodendroglia (McCall et al., 2014) can still bear some problems, bioengineered extracellular vesicles are emerging as delivery vehicles for such therapeutic agents (Wiklander et al., 2019). Another challenge is to identify the right therapeutic intervention window. Regenerative agents delivered too late during disease course could be useless since axons might be already irreversibly committed to degeneration. Other issues are the proper design of clinical trials (e.g., correct stratification of patients) and the development of appropriate outcome measures, which are both critical to demonstrate the efficacy of a remyelinating drug (Ontaneda et al., 2015). Importantly, the complex pathophysiology of progressive MS suggests that combination therapies targeting different processes would represent the “ideal” therapeutic approach, as anticipated for GPR17 (Coppolino et al., 2018; Lecca et al., 2020). Although several issues still remain to be addressed, the emerging and promising developments in clinical remyelination therapy (Plemel et al., 2017) raise hope for having soon new therapeutic options for progressive MS.

## Author Contributions

All authors listed have made a substantial, direct, and intellectual contribution to the work and approved it for publication.

## Funding

Supported by FISM—Fondazione Italiana Sclerosi Multipla—cod. 2017/R/1 to MPA and financed or co-financed with the ‘5 per mille’ public funding.

## Conflict of Interest

The authors declare that the research was conducted in the absence of any commercial or financial relationships that could be construed as a potential conflict of interest.
